# Investigating Urinary Complications in Young Infant Surgical Patients with Indwelling Epidural Catheters: A Retrospective Cohort Study

**DOI:** 10.3390/children12070833

**Published:** 2025-06-24

**Authors:** Mihaela Visoiu, Dahye Park, Erin E. Simonds, Senthilkumar Sadhasivam

**Affiliations:** 1 Department of Anesthesiology and Perioperative Medicine, University of Pittsburgh School of Medicine, UPMC Children’s Hospital of Pittsburgh, Pittsburgh, PA 15224, USA; sadhasivams@upmc.edu; 2 Department of Anesthesiology and Perioperative Medicine, UPMC Presbyterian, Pittsburgh, PA 15213, USA; parkd6@upmc.edu; 3 Department of Pediatric Chronic Pain, UPMC Children’s Hospital of Pittsburgh, Pittsburgh, PA 15224, USA; cummingsee3@upmc.edu

**Keywords:** infants, continuous epidural analgesia, postoperative urinary retention, urinary tract infections, opioid, Foley catheter management

## Abstract

Background/Objectives: Continuous epidural analgesia (CEA) is commonly used to manage postoperative pain in young infants. However, it can impair bladder function, leading to postoperative urinary retention (POUR) and necessitating Foley catheter placement, which carries a risk of urinary tract infection (UTI). Limited research exists on the frequency of POUR and UTIs and factors influencing optimal Foley catheter management in this population. Methods: A retrospective chart analysis conducted at UPMC Children’s Hospital of Pittsburgh included 103 infants who had surgery with CEA. The patients were assigned to Group A (Foley catheter removed before epidural discontinuation), Group B (Foley catheter removed after epidural discontinuation), and Group C (no Foley catheter placement). Data collected included demographics, details regarding urinary complications, epidural analgesia, pain management, and Foley catheter management. Results: The median/IQR age was 8 weeks (0.71–13.29), and the weight was 3.01 (2.55–3.52) kg. POURs occurred shortly after surgery in two (1.9%) infants with no initial Foley catheter placement (*p* = 0.101). Two (1.9%) infants in Group B developed a UTI (*p* = 0.327). A total of 10 (9.7%) (Groups A and B) had a preexisting urologic condition (*p* = 0.040). Common surgeries included exploratory laparotomy with bowel resection (34%) and stoma closure (28.2%). The epidural catheter was discontinued on postoperative day 3 (median) (*p* = 0.587). Total opioid administration, median/IQR (MME mg/kg), was significantly higher in Group B (1.7/0.6–3.8) and Group A (0.7/0.3–1.8) compared to Group C (0.6/0.3–1.1) (*p* = 0.029). Conclusions: No POUR occurred when the Foley catheter was removed before the epidural was discontinued. UTIs occurred when the Foley catheter remained after epidural discontinuation. Our findings highlight the importance of individualized assessment for urinary catheter placement and early removal in young infants receiving CEA.

## 1. Introduction

Continuous epidural analgesia (CEA) is a widely utilized regional anesthesia technique for postoperative pain management in neonates and young infants undergoing surgical procedures [1,2,3]. It offers superior analgesia, reduces opioid consumption, and minimizes opioid-related side effects compared to systemic analgesia [2,4].

Epidural analgesia, however, can impair autonomic nervous system control of the bladder, leading to detrusor muscle dysfunction, diminished sensation of bladder fullness, and postoperative urinary retention (POUR) [5,6]. To prevent such complications, a Foley catheter is placed and maintained until the epidural catheter is discontinued. Unfortunately, Foley catheter placement increases the risk of urinary tract infection (UTI) [7,8,9]. Both POUR and UTI can prolong hospital stays and increase morbidity [8,10].

Neonates and young infants may not need Foley catheter placement, as some can still void spontaneously with an epidural catheter. Potential factors that could contribute to postoperative urinary retentions are gender, associated urologic comorbidities, the type of surgical procedure performed, the epidural catheter tip location, the type and amount of epidural infusion, and the amount of opioid administered [5,6,8].

Identifying factors contributing to POUR is critical in this population to determine the best timing for inserting and removing Foley catheters. Understanding the frequency of POUR and UTI is vital for establishing evidence-based guidelines when CEA is employed for postoperative pain relief in neonates and young infants.

To address these concerns, we performed a retrospective study involving 103 infants admitted to the Neonatal Intensive Care Unit (NICU) after undergoing various surgical procedures and receiving CEA for pain control. This study aims to investigate the relationship between continuous epidural analgesia, Foley catheter placement, and urinary complications (POUR and UTI) and identify risk factors influencing the need for and duration of Foley catheter placement.

## 2. Materials and Methods

### 2.1. Study Design

This research was a retrospective, descriptive analysis at UPMC Children’s Hospital of Pittsburgh, focusing on infants admitted to the Neonatal Intensive Care Unit (NICU) after surgical procedures. The study period extended from July 2018 to September 2024. Approval from the Institutional Review Board (IRB) was obtained before data collection, with a waiver of informed consent due to the study’s retrospective nature (STUDY20050148 approved on 1 June 2020).

### 2.2. Study Population

The study cohort included 103 infants (44 females and 59 males) whose ages at birth ranged from 22 weeks and 3 days to 41 weeks and 5 days, who underwent surgical interventions requiring postoperative CEA. The corrected gestational age at the time of surgery ranged from 31 weeks and 5 days to 54 weeks and 5 days. The study included four patients who were deceased at the time of the chart review, which was conducted after September 2024.

### 2.3. Data Collection

Data was collected retrospectively from electronic medical records and categorized as follows:

Demographic information: gestational age at birth, gender, weight at the time of surgery, and presence of preexisting urologic conditions.

Anesthetic Data: American Society of Anesthesiologists (ASA) physical status classification, total anesthesia duration, epidural catheter insertion, tip location, and intraoperative opioid administration.

Surgical Data: Type of surgical procedures and documentation of Foley catheter insertion during surgery.

Postoperative Data: Postoperative opioid and local anesthetic administration, the timing of Foley catheter removal, the timing of epidural catheter removal, the occurrence of POUR, incidence of UTIs, requirement for intermittent catheterization following Foley catheter removal, requirement for Foley catheter re-insertion, documentation of urine leakage around the Foley catheter.

### 2.4. Surgical Procedure Performed

A total of 103 surgical cases were analyzed in this study. The distribution of surgical procedures was as follows: Ileostomy closures (*n* = 32), Exploratory laparotomies (*n* = 14), Bowel resections (*n* = 12), Jejunostomy closures (*n* = 6), Duodenal atresia repairs (*n* = 8), Tracheoesophageal fistula repairs (*n* = 9), Congenital diaphragmatic hernia repairs (*n* = 3), Omphalocele closures (*n* = 2), Nissen fundoplications (*n* = 2), Neuroblastoma resection (*n* = 1), Pheochromocytoma resection (*n* = 1), and Ladd’s procedure for intestinal malrotation (*n* = 1).

### 2.5. Details of Anesthesia and Epidural Catheter Placement

All surgical interventions were performed under general anesthesia with endotracheal intubation. Anesthetic medications were not standardized and were determined based on the attending anesthesiologist’s judgment. All neuraxial catheters were placed while the infant was under general anesthesia. In 70 cases, a caudal approach was used to advance the catheter to the required thoracic level, from T4 to T12. The epidural catheters were placed when moderate to severe pain was anticipated after surgery, and when both the neonatologist and surgeon believed that the catheter would aid in pain management and facilitate extubation postoperatively. Catheters were not placed if the patient was coagulopathic, had a systemic infection, or displayed abnormal spine findings at the sacral, lumbar, or thoracic levels.

The remaining 33 catheters were placed using an intervertebral approach (8 lumbar and 25 thoracic). Intraoperatively, the patients received fentanyl (administered to 78 infants), methadone (1 infant), acetaminophen (47 infants), dexmedetomidine (21 infants), and ropivacaine (0.1–0.2%) delivered via the epidural catheter.

### 2.6. Foley Catheter Management Protocol

Our institution’s policy is to place a Foley catheter in all patients with an epidural catheter, with the Foley catheter typically removed a few hours after the epidural catheter is discontinued. In some cases, the surgical team, pain management team, and neonatologists choose not to place or remove the Foley catheter before removing the epidural catheter. If an infant with an epidural has absent or minimal urinary output, a straight catheterization is performed to evaluate urinary retention or the need for additional fluids to optimize fluid status. A Foley catheter is preferred over intermittent catheterization to reduce the risk of urinary tract infections and is also indicated when the infant exhibits generalized edema, is oversedated, or is receiving muscle relaxants.

### 2.7. Urinary Outcomes

POUR was identified by an inability to void postoperatively within 8 h, requiring catheterization or re-insertion of a Foley catheter due to a full bladder, with criteria ensuring consistent diagnosis across patients [8]. UTI was defined following CDC 2024 guidelines [11]. A UTI in patients aged 1 year or younger is diagnosed when all of the following are met: (1) the patient is ≤1 year old and may or may not have an indwelling urinary catheter (IUC); if the IUC was in place for more than two consecutive days and still in place or removed the day before symptom onset, the case is considered CA-UTI (catheter-associated UTI); otherwise, it is a non-catheter-associated UTI; (2) the patient has at least one symptom without another known cause—fever > 38 °C, hypothermia < 36 °C, apnea, bradycardia, lethargy, vomiting, or suprapubic tenderness; and (3) a urine culture shows no more than two organisms, with at least one being a bacterium at ≥10^5^ CFU/mL. All elements must occur within the infection window period [11]. Due to the patient’s very young age, we reviewed the records up to 10 days after Foley catheter removal for any positive culture and antibiotic treatment-confirmed UTI cases. We tried to differentiate between CA-UTI and non-catheter-associated UTI. The UTI rate was defined as all UTI cases/total patients. The CA-UTI rate per 1000 urinary catheter days was determined by dividing the number of CA-UTIs by the total number of catheter days and then multiplying the result by 1000 [11].

We also examined urine leakage around the Foley catheter during our chart review. This could indicate that the patient will be able to urinate in the absence of a Foley catheter despite CEA.

### 2.8. Postoperative Pain Management Medications

The standard protocol for epidural catheter-based pain management involves keeping the epidural catheter in place until postoperative day 3. The neonatologists wrote postoperative pain plans, except for the local anesthetic infusion that was ordered by the pain management team. The epidural infusions used included ropivacaine (0.05%, 0.1%, 0.2%) with clonidine (1 or 2 mcg/mL) for 19 infants. Postoperative medications administered included morphine (98 infants), fentanyl (9 infants), acetaminophen (88 infants), dexmedetomidine (3 infants), lorazepam, and midazolam (33 infants).

Morphine, given either as needed doses or infusion, was frequently the primary medication used for pain management and sedation. Fentanyl was occasionally used as a substitute for morphine. None of the patients received ketorolac. Postoperative medication administration was documented at 24-h intervals until the epidural catheter was discontinued.

### 2.9. Study Aims

We hypothesized that young infants undergoing surgical procedures who received an epidural catheter for postoperative analgesia might have trouble urinating and develop postoperative urinary retention if a Foley catheter was not placed or was removed before the epidural catheter was discontinued.

The main objective of this study was to identify the incidence of POUR in the absence of a Foley catheter or after the Foley catheter was removed.

Additionally, we hypothesized that the presence of a Foley catheter could increase the incidence of urinary tract infections. The secondary objective was to investigate the incidence of UTIs in infants who underwent epidural catheter placement for postoperative pain management, comparing those who received a Foley catheter to those who did not.

The third objective was to identify the factors that impact the necessity and duration of Foley catheter placement, such as gender, associated urologic comorbidities (acute kidney injury and congenital urogenital abnormalities), the type of surgical procedure performed, the epidural catheter tip location, the type and amount of epidural infusion, and the amount of opioid administered.

To investigate these aims, infants were divided into three groups based on the presence or absence of a Foley catheter and its removal relative to epidural management: Group A (the Foley catheter was placed but removed before the epidural was discontinued), Group B (the Foley catheter was placed but removed after the epidural was discontinued), and Group C (no Foley catheter placement while the epidural was in situ). If the infant did not have a Foley catheter at the end of surgery but required one later, the infant was included in a group with a Foley catheter, A or B.

### 2.10. Statistical Analysis

Descriptive statistics were computed using medians and interquartile ranges (IQR) for continuous data and counts and percentages for categorical data. Differences between the three treatment groups (A, B, and C) were assessed for continuous data using the Kruskal–Wallis test. In contrast, categorical differences were analyzed using Chi-squared tests and Fisher’s exact tests. Data visualization was performed using histograms and box plots.

Opioid administrations were converted to milligram morphine equivalents (MMEs) (mg/kg). Missing values were excluded from all denominators and statistical analyses. *p*-values < 0.05 were considered significant. Data management and analysis were conducted using R software (version 4.3.3, R Core Team, 2024).

## 3. Results

### 3.1. Demographics

A total of 103 infants were included in this study and divided into three groups: Group A (*n* = 33), Group B (*n* = 36), and Group C (*n* = 34).

Table 1 presents the study population’s baseline characteristics. The median gestational age at birth tends to be slightly lower in Group C (31.2 weeks) compared to Groups A and B (both 36.7 weeks) (*p* = 0.051). None of the infants in Group C had a preexisting urologic procedure (*p* = 0.040). A total of 10 preexisting urologic conditions were identified among the study participants, with an equal distribution of 5 cases in Group A and 5 cases in Group B. The identified conditions included left renal pelvicaliectasis, bilateral vesicoureteral reflux, a teratoma involving the left kidney leading to surgical removal, duplicated left collecting system, mild left pelvocaliectasis, left kidney atrophy, hydronephrosis, and acute kidney injury (AKI) in three cases.

### 3.2. Urinary Complications Outcome

Postoperative urinary retention occurred in 2 of 103 patients (1.9%) (*p* = 0.101). Neither infant had a Foley catheter placed after surgery, but both required Foley catheter placement in the first 8 h after surgery. The NICU team decided to insert the Foley catheter. Both patients had their Foley catheters removed before the epidural was discontinued, and they were assigned to group A.

Two patients (1.9%) had urinary tract infections (UTIs), both in the group with the Foley catheter removed after the epidural was discontinued (Group B) (*p* = 0.327). One infant developed a catheter-associated urinary tract infection (Table 2). CA-UTI rate among catheterized patients was 1 out of 69 (1.4%), with CA-UTI rate per 1000 urinary catheter days of 5.9.

Another infant had a history of urologic condition and developed a non-catheter-related UTI. Both infants required antibiotic treatment; however, no UTI-related complications, such as pyelonephritis or urosepsis, were recorded.

Urine leakage around the Foley catheter was observed in 12 patients (17.9%), with similar distribution in Groups A (16%) and B (19%) *p* = 0.761).

### 3.3. Surgical Procedures Performed

Table 3 presents the distribution of surgical procedures across groups. There is no statistical difference in the surgical procedures performed. Procedures combining circumcision with other surgeries (e.g., laparotomy and stoma closure) were relatively infrequent, with no significant group differences.

### 3.4. Epidural Catheter Characteristics

#### 3.4.1. Eoidural Catheter Location

There is no statistical difference between the epidural catheter insertion sites (*p* = 0.692). All the epidural catheter tips were located at the thoracic level, 47 between the T4 and T9 vertebral levels, 26 between the T10 and T12 vertebral levels, and 30 at the unspecified thoracic level.

The postoperative day of epidural catheter removal was similar across all groups (median of 3 days), with no statistically significant difference (*p* = 0.587).

#### 3.4.2. Epidural Ropivacaine Infusion Concentration

Postoperatively, ropivacaine infusion was administered via the epidural catheter at three concentrations: 0.05%, 0.1%, and 0.2%, with no difference between the groups (Table 4) (*p* = 0.974).

#### 3.4.3. Total Epidural Ropivacaine Administration

The total median ropivacaine dose administered via the epidural catheter, including intraoperative bolus and postoperative infusion, was 17.57 mg/kg (IQR: 15.08–21.24) across all groups. No statistically significant differences were noted among the groups (*p* = 0.0754).

### 3.5. Opioid Administration

#### 3.5.1. Opioid Administration During and Following Surgery

**Figure 1:**
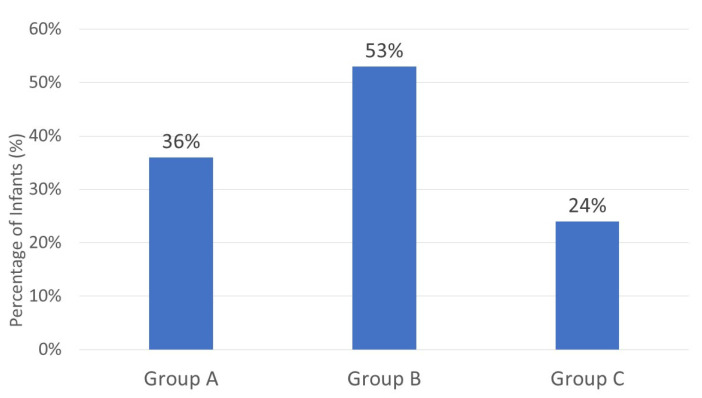
Postoperative opioid infusion. % shows the percentages of infants from each group. Postoperative opioid infusion was required in 53% of Group B patients, representing a statistically significant difference among the groups. Group A (Foley catheter was removed before the epidural was discontinued), Group B (Foley catheter was removed after the epidural was discontinued), and Group C (no Foley catheter placement).

Intraoperative opioid use (fentanyl) was reported in 80 infants (77.7%): 27 infants (82%) in Group A, 30 infants (83%) in Group B, and 23 infants (68%) in Group C (*p* = 0.253). Postoperative opioid infusion, involving morphine (35 infants) and fentanyl (4 infants), showed significant variation among groups: 12 infants in Group A, 19 in Group B, and 8 in Group C (*p* = 0.042) (Figure 1). It refers to patients who received a continuous infusion of opioids for pain management following surgery, in addition to the CEA.

#### 3.5.2. Total Opioid Administration in MME

There is no statistically significant difference across the groups in the total opioid administration (intravenous MME mg/kg) intraoperatively (*p* = 0.072) or postoperatively (*p* = 0.088) (Table 5).

**Figure 2:**
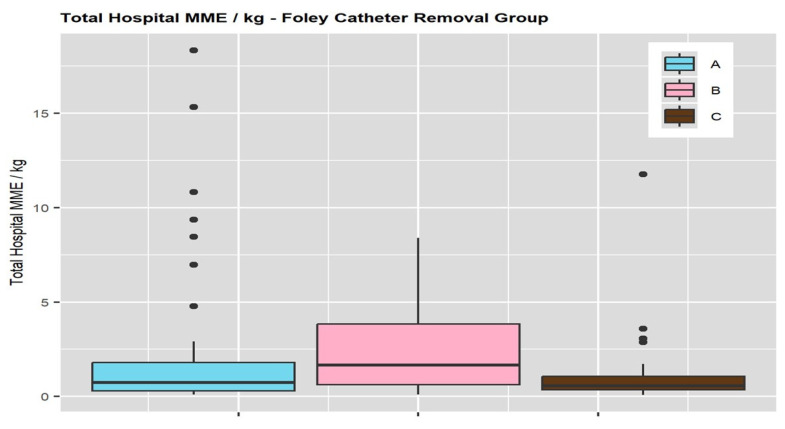
Total opioid administration was reported as intravenously, MME (mg/kg). Group A (Foley catheter was removed before the epidural was discontinued), Group B (Foley catheter was removed after the epidural was discontinued), and Group C (no Foley catheter placement).

Total opioid administration intraoperatively and postoperatively, median/IQR (MME mg/kg), was significantly higher in Group B (1.7/0.6–3.8) and Group A (0.7/0.3–1.8) compared to Group C (0.6/0.3–1.1) (*p* = 0.029) (Figure 2). Total Opioid Administration (Intraoperative and Postoperative) encompasses both bolus doses and continuous opioid infusions given during and after surgery, throughout the CEA period, until the epidural was discontinued.

## 4. Discussion

Our study included 103 infants who underwent surgery with indwelling thoracic epidural catheters for postoperative pain management. We assessed the incidence of urinary complications—specifically postoperative urinary retention (POUR) and urinary tract infections (UTIs)—by dividing patients into three groups based on the presence of a Foley catheter and the timing of its removal in relation to epidural catheter discontinuation.

Previous studies have reported varying POUR rates. Gupta et al. [6] found a 4.4% incidence of POUR, while Mansfield et al. [12] described it as a minor adverse effect. Cathleen et al. [13] found that 9.2% of children with cerebral palsy undergoing hip or lower limb surgeries, with or without epidural analgesia, required intermittent catheterization after Foley catheter removal, with 3.3% needing re-catheterization. Yotam et al. reported a 5% rate of Foley catheter re-insertion in 57 pediatric patients undergoing lower extremity orthopedic surgery with epidural analgesia [14].

Our study identified POUR in 2 out of 103 patients (1.9%). Neither of these patients had a Foley catheter placed immediately after surgery; both later developed urinary retention that required re-catheterization. No cases of POUR occurred in infants whose Foley catheters were removed either before or after discontinuing the epidural, suggesting that POUR is relatively uncommon in this population.

Several studies have also explored the incidence of UTIs in pediatric surgical patients. A large-scale analysis of 369,176 pediatric cases from the NSQIP Pediatric database (2012–2016) reported a 0.5% UTI rate [10]. Similarly, Walker et al. found a very low postoperative infection rate, including UTIs, in a large cohort of pediatric patients receiving regional anesthesia [15].

Hospital records from 2014 to 2020 revealed 62 catheter-associated UTI (CA-UTI) cases in the pediatric intensive care unit (PICU), with an incidence of 7.2 infections per 1000 catheter days [7]. Samraj et al. reported 5751 PICU patients with indwelling urinary catheters, yielding a utilization ratio of 0.38. With 16,971 total catheter days, they observed a CA-UTI rate of 3.0 per 1000 catheter days [16]. Our findings (5.9 per 1000 catheter days) were comparable. However, we focused specifically on neonates and young surgical infants—a group underrepresented in research on urinary complications related to epidural catheter use.

Current research and guidelines emphasize careful Foley catheter management to reduce infection risk, particularly in patients receiving epidural analgesia [8,10]. For example, the Surgical Care Improvement Project (SCIP) recommends removing Foley catheters by postoperative day 1 or 2 [17]. However, studies in adult populations suggest that early removal during epidural use may increase POUR risk, making removal after discontinuation preferable [18,19,20]. Notably, there are no guidelines for Foley catheter use in young infants.

Our study found low rates of POUR with no significant impact from Foley catheter removal timing. Early removal before epidural discontinuation appears safe, though further research is needed to validate these results.

Several factors influence POUR, including anesthetic, surgical, and patient-related variables. Opioids can suppress bladder signaling, increasing the risk of retention. Other known risk factors include older age, male sex, urologic conditions, longer surgeries, and excessive IV fluids [8]. To explore potential contributors to POUR and Foley catheter need, we analyzed patient characteristics, preexisting urologic issues, surgical procedures, epidural insertion level, and tip location, as well as ropivacaine and opioid use.

We found that opioid use was more prevalent in infants who had Foley catheters placed, and opioid consumption was highest in those whose catheters were removed after epidural discontinuation. This suggests a potential link between opioid use and the need for catheterization in young infants receiving epidural analgesia. Infants with preexisting urologic conditions, such as acute kidney injury or congenital abnormalities, also appeared more likely to require catheter placement. Further investigation is needed to clarify these associations.

Additionally, 17.9% of infants experienced urine leakage around the Foley catheter, suggesting that routine catheter placement may not be necessary for all young surgical patients with epidural catheters. Avoiding Foley catheter placement may be appropriate for infants without preexisting urologic conditions, particularly when the epidural catheter tip is at the thoracic level and opioid use is expected to be low. This is especially relevant for associated procedures like circumcision, where a catheter could interfere with wound care. If catheterization is performed, early removal may be considered for infants receiving thoracic analgesia who are voiding around the catheter and have minimal opioid requirements.

### Limitations

While our study provides valuable information about urinary complications in young infants with indwelling epidural catheters, several limitations must be acknowledged. (1) This study included 103 infants from a single institution, which may limit its generalizability. The retrospective design limits control over confounding variables, such as Foley catheter placement, opioid administration, and epidural discontinuation. (2) The absence of a standardized protocol for Foley catheter placement and removal could have influenced outcomes. (3) Diagnosing POUR in neonates and young infants is challenging due to their limited ability to express discomfort, potentially leading to underreporting of this complication. (4) We lacked data on prior surgical procedures and previous opioid exposure, both of which could significantly affect postoperative opioid requirements and pain scores. Opioid use was more common in infants whose Foley catheters were removed after epidural discontinuation. However, its precise relationship with POUR remains unclear. (5) This study’s low incidence of complications may limit our ability to draw definitive conclusions about the risk factors or outcomes associated with urinary retention and infections. A larger sample size or a study with a higher complication rate is needed to understand these associations better. (6) The findings may not be generalized to infants undergoing non-surgical procedures or those managed with alternative pain control strategies.

## 5. Conclusions

In conclusion, our study provides valuable insights into the incidence of postoperative urinary complications, such as postoperative urinary retention (POUR) and urinary tract infections (UTIs), in 103 young infants undergoing surgery with indwelling thoracic epidural catheters. We observed two cases of POUR and two cases of UTIs, with no significant differences based on the timing of urinary catheter removal relative to the discontinuation of the epidural catheter. UTIs occurred when the urinary catheter remained in place after the epidural catheter was discontinued.

Routine urinary catheterization may not be necessary for all infants, especially those with thoracic-level epidural catheter tips, expected low opioid use, and no preexisting urologic conditions. Our study suggests that early Foley catheter removal may be appropriate for selected infants with thoracic epidural analgesia, especially those with minimal opioid use and urine leakage around the catheter. Further prospective studies are warranted to establish individualized strategies for urinary catheter management in young infants receiving continuous epidural analgesia. Our findings underscore the need for additional research to inform clinical guidelines and improve perioperative care in this population.

## Figures and Tables

**Table 1:** The demographics of the patient population and the specific surgical, anesthesia, and epidural attributes. The variables were reported as median and interquartile range (IQR) or percentage. N represents the number of total patients, *n* represents the number of patients from each group, and % shows percentages from each group. The American Society of Anesthesiologists’ physical classification system, ASA, is a standard method used to evaluate a patient’s medical comorbidities and help predict perioperative risk. It consists of six ordinal scoring categories to communicate a general assessment of underlying physiological status, ranging from entirely healthy (ASA Class I) to brain-dead awaiting organ procurement for donation (ASA Class VI). It had high validity and predictive accuracy for postoperative mortality. Preexisting urologic conditions investigated in our article included acute kidney injury and congenital urogenital abnormalities. The day of epidural removal is the postoperative day when the epidural is discontinued. Group A (Foley catheter was removed before the epidural was discontinued), Group B (Foley catheter was removed after the epidural was discontinued), and Group C (no Foley catheter placement).

Variable	Total Median Median (IQR) N = 103	Group A Median (IQR) *n* = 33	Group B Median (IQR) *n* = 36	Group C Median (IQR) *n* = 34	*p*-Value
Gestational age at birth (weeks)	34.86 (26.43–37.71)	36.7 (30.9–38.4)	36.7 (30.9–38.4)	31.2 (25.4–36.6)	0.051
Weeks of life at the surgery	8 (0.71–13.29)	2 (0.4–11.1)	2 (0.4–11.1)	9.6 (1.2–15.5)	0.145
Weight (kg)	3.01 (2.55–3.52)	3.1 (2.6–3.6)	3.1 (2.6–3.6)	3 (2.5–3.5)	0.743
Female	44 (42.7%)	14 (42%)	16 (44%)	14 (41%)	0.962
Male	59 (57.3%)	19 (58%)	20 (56%)	20 (59%)	
Preexisting Urologic Condition	10 (9.7%)	5 (15%)	5 (14%)	0 (0%)	0.040
ASA status	3 (3–3)	3 (3–3)	3 (3–3)	3 (3–3)	0.036
Anesthesia length (minutes)	295 (249.5–354)	281 (235–358)	281 (235–358)	261 (230.2–332.8)	0.006
Surgery length (hours)	2.82 (2.16–3.6)	2.8 (2–3.5)	2.8 (2–3.5)	2.4 (2.1–3.3)	0.044
The day of epidural removal	3 (3–3)	3 (3–4)	3 (3–4)	3 (3–3)	0.587

**Table 2:** Urinary complications. N represents the number of total patients, *n* represents the number of patients from each group, and % shows percentages from each group. Group A (Foley catheter was removed before the epidural was discontinued), Group B (Foley catheter was removed after the epidural was discontinued), and Group C (no Foley catheter placement).

Variable	Total N = 103 (%)	Group A *n* = 33 (%)	Group B *n* = 36 (%)	Group C *n* = 34 (%)	*p*-Value
Postoperative urinary retention	2 (1.9%)	2 (6%)	0 (0%)	0 (0%)	0.101
Urinary tract infection	2 (1.9%)	0 (0%)	2 (6%)	0 (0%)	0.327

**Table 3:** The group’s surgical procedure. N represents the number of total patients, *n* represents the number of patients from each group, and % shows percentages from each group. Group A (Foley catheter was removed before the epidural was discontinued), Group B (Foley catheter was removed after the epidural was discontinued), and Group C (no Foley catheter placement).

Variable Value	Total N = 103 *n* (%)	Group A N = 33 *n* (%)	Group B N = 36 *n* (%)	Group C N = 34 *n* (%)	*p*-Value
Exploratory Laparotomy Bowel Resection	35 (34%)	13 (39%)	15 (42%)	7 (21%)	0.066
Exploratory Laparotomy, Bowel Resection, and Circumcision	8 (7.8%)	2 (6%)	1 (3%)	5 (15%)	
Thoracic Procedure	13 (12.6%)	7 (21%)	5 (14%)	1 (3%)	
Thoracic Procedure and Circumcision	1 (1%)	1 (3%)	0 (0%)	0 (0%)	
Stoma Closure	29 (28.2%)	5 (15%)	10 (28%)	14 (41%)	
Stoma Closure and Circumcision	10 (9.7%)	2 (6%)	4 (11%)	4 (12%)	
Other	6 (5.8%)	3 (9%)	1 (3%)	2 (6%)	
Other and Circumcision	1 (1%)	0 (0%)	0 (0%)	1 (3%)	

**Table 4:** Postoperative epidural ropivacaine infusion concentration. N represents the number of total patients, *n* represents the number of patients from each group, and % shows percentages from each group. Group A (Foley catheter was removed before the epidural was discontinued), Group B (Foley catheter was removed after the epidural was discontinued), and Group C (no Foley catheter placement).

Ropivacaine Concentration	Total N = 103 (%)	Group A *n* = 33 (%)	Group B *n* = 36 (%)	Group C *n* = 34 (%)	*p*-Value
0.05%	1 (1%)	0 (0%)	1 (3%)	0 (0%)	0.974
0.1%	91 (88.3%)	29 (88%)	31 (86%)	31 (91%)	
0.2%	11 (10.7%)	4 (12%)	4 (11%)	3 (9%)	

**Table 5:** Perioperative opioid administration reported as intravenously, MME (mg/kg). N represents the number of total patientsGroup A (Foley catheter was removed before the epidural was discontinued), Group B (Foley catheter was removed after the epidural was discontinued), and Group C (no Foley catheter placement); (IQR) is the interquartile range.

Variable (MME; mg/kg)	Total Median (IQR) N = 103	Group A Median (IQR) N = 33	Group B Median (IQR) N = 36	Group C Median (IQR) N = 34	*p*-Value
Total Intraoperative Opioid Use	0.18 (0.05–0.33)	0.2 (0.1–0.3)	0.2 (0.1–0.5)	0.1 (0–0.3)	0.072
Total Postoperative Opioid Use	0.49 (0.17–1.71)	0.5 (0.1–1.7)	1.4 (0.3–3.7)	0.4 (0.2–0.9)	0.088

## Data Availability

UPMC Children’s Hospital, Department of Anesthesia archived dataset analyzed.
